# Treatment patterns and effectiveness of patients with multiple myeloma initiating Daratumumab across different lines of therapy: a real-world chart review study

**DOI:** 10.1186/s12885-021-08881-7

**Published:** 2021-11-12

**Authors:** Shebli ATRASH, Philippe THOMPSON-LEDUC, Ming-Hui TAI, Shuchita KAILA, Kathleen GRAY, Isabelle GHELERTER, Marie-Hélène LAFEUILLE, Patrick LEFEBVRE, Adriana ROSSI

**Affiliations:** 1 Levine Cancer Institute, Charlotte, NC USA; 2 Analysis Group, Inc, 1190 avenue des Canadiens-de-Montréal, Deloitte Tower, Suite 1500, Montreal, QC, H3B 0G7 Canada; 3 Janssen Scientific Affairs, LLC, Horsham, PA, USA; 4 Division of Hematology and Medical Oncology, Weill Cornell Medicine, New York, NY USA

**Keywords:** Multiple myeloma, Daratumumab, Overall response rate, Progression-free survival, Time to next treatment, Drug therapy, Real-world

## Abstract

**Background:**

Daratumumab, a CD38 monoclonal antibody, has demonstrated efficacy as monotherapy and combination therapy across several indications, both among newly-diagnosed and refractory patients with multiple myeloma (MM). However, there is limited evidence on treatment patterns and effectiveness of daratumumab in the real-world setting, particularly in first line (1 L). This study aimed to describe real-world treatment patterns and clinical outcomes among patients initiating daratumumab across different lines of therapy.

**Methods:**

A retrospective chart review of adult patients with MM initiating daratumumab between November 2015 and March 2021 was conducted at two clinical sites in the United States. De-identified patient-level data were abstracted in an electronic case report form. Patient characteristics and treatment patterns were described. Clinical outcomes including overall response rate (ORR), progression-free survival, and time to next line of therapy were reported using descriptive statistics and stratified by line of therapy (1 L, second line [2 L] or third line or later [3 L+]). A sub-group analysis evaluated treatment patterns and ORR among patients re-treated with daratumumab.

**Results:**

A total of 299 patients were included in the study (mean age: 68 years; 55% male). Among them, 26 were 1 L patients, 66 were 2 L patients, and 207 were 3 L+ patients; 110 patients (36.8%) received a stem cell transplant prior to daratumumab initiation. The mean duration of follow-up was 10 months among 1 L patients and 19 months among 2 L and 3 L+ patients. Patients who initiated daratumumab in 1 L had a 100% ORR, while those initiating in 2 L and 3 L+ had an ORR of 78.8 and 65.2%, respectively. Among re-treated patients, ORR was 66.7% during the first treatment segment, and 52.9% during the second treatment segment. Kaplan-Meier rates of progression-free survival at 12 months were 89.9, 75.2, and 53.1% among patients who initiated daratumumab in 1 L, 2 L, and 3 L+, respectively. Kaplan-Meier rates of time to next line of therapy at 12 months were 94.1, 73.4, and 50.0% among patients who initiated daratumumab in 1 L, 2 L, and 3 L+, respectively.

**Conclusions:**

These findings suggest that daratumumab-based regimens are an effective treatment option across all lines of therapy, with highest response rate in 1 L.

**Supplementary Information:**

The online version contains supplementary material available at 10.1186/s12885-021-08881-7.

## Background

Multiple myeloma (MM) is characterized by the accumulation of neoplastic plasma cells in the bone marrow [1]. The incidence of MM is expected to account for 1.8% of all new cancer cases and for 2.0% of all cancer deaths in the United States (US) in 2021 [2]. Between 2011 and 2017, the average five-year survival rate of patients diagnosed with MM was 55.6% [2].

Daratumumab is a human monoclonal antibody targeting CD38 approved for the treatment of MM [3]. Daratumumab monotherapy was first approved by the Food and Drug Administration (FDA) for patients with relapsed and/or refractory MM who have received at least three prior treatments (including at least one proteasome inhibitor and an immunomodulatory agent) in November 2015 [4–6]. Daratumumab was subsequently approved for the treatment of MM in combination with lenalidomide and dexamethasone, [7] or bortezomib and dexamethasone [8] among patients who have received at least one prior therapy and in combination with pomalidomide and dexamethasone among patients with relapsed or refractory MM [9]. In May 2018, daratumumab was approved for use in front line among patients who are ineligible for autologous stem cell transplant (ASCT) [10] and among ASCT-eligible patients in September 2019 [11].

While the safety and efficacy of daratumumab in front line and later lines has been well documented in clinical trials, real-world insights on treatment patterns and outcomes among patients with MM initiated on daratumumab, including among patients treated and re-treated with daratumumab, [12] are limited. In light of the rapidly-evolving treatment landscape in MM, [13] there is a need to understand real-world outcomes associated with daratumumab among patients with newly-diagnosed and relapsed or refractory MM. Therefore, the goal of this study was to describe the real-world treatment patterns and clinical outcomes of adult patients with MM receiving daratumumab across different lines of therapy.

## Methods

### Data source

A retrospective study design using data from electronic medical records (EMR) and medical charts was employed. De-identified data were retrieved from two clinical sites, Levine Cancer Institute (Atrium Health) and Weill Cornell Medicine. Chart abstraction was conducted between July 2020 and February 2021. Charts were randomly selected among daratumumab-treated patients at each institution using an algorithm based on the first letter of the patients’ last name. Structured and unstructured data were entered into an electronic case report form (eCRF). This study was reviewed and approved by the institutional review board of each site involved (Atrium Health Institutional Review Board and Weill Cornell Medicine Institutional Review Board) prior to the initiation of data retrieval.

### Patient inclusion

Patients were included if they had a confirmed diagnosis of MM by the treating physician in their patient record, were at least 18 years old at the time of daratumumab initiation, and had complete treatment history available between MM diagnosis and daratumumab initiation. Patients who accessed daratumumab through interventional clinical trials were excluded. Patients were followed from the initiation of daratumumab until death, loss to follow-up, or date of chart abstraction completion, whichever occurred first.

### Study measures

Patient demographic characteristics were reported, including the age at time of daratumumab initiation, time between MM diagnosis and daratumumab initiation, sex, race, and primary insurance plan type. Patient clinical characteristics reported included MM stage at diagnosis based on the Revised International Staging System (R-ISS) for multiple myeloma [14], cytogenetic profile as of daratumumab initiation (high risk defined as del(17p), t(4;14) or t(14;16)), refractory disease on treatments prior to initiating daratumumab, and the year of daratumumab initiation.

Treatment patterns were described for patients’ first daratumumab-based regimen, including the number of lines of therapy and individual regimens received prior to daratumumab initiation (as per physician notes), the regimen, whether patient received a stem cell transplant prior to initiating the regimen, the regimen type (i.e., induction therapy, conditioning therapy, consolidation therapy, maintenance therapy post-stem cell transplant, bridging therapy), and the length of the regimen.

Treatment response to the first daratumumab-based regimen was reported, as per physician notes and according to the criteria from the International Myeloma Working Group [15] (i.e., stringent complete response, complete response, very good partial response, partial response, minimal response, stable disease, progressive disease, clinical relapse, other). Overall response was defined as “partial response” or better among patients with known response rate. Response rate of “very good partial response” or better among patients with known response rate was also reported.

Clinical outcomes also included progression-free survival (PFS),time to next line of therapy, and overall survival. Disease progression was defined as a record of discontinuation due to progressive disease, progressive disease as a patient’s best response to a treatment regimen, or death and was measured from the initiation of daratumumab onward. Patients not experiencing disease progression were censored at the initiation of a subsequent line of therapy or the end of follow-up, whichever occurred first. Time to next line of therapy was defined as the time between the initiation of the first daratumumab-based regimen and the initiation of the following line of therapy. Patients who did not initiate a subsequent line of therapy were censored at the end of follow-up. Overall survival was defined as the time between the initiation of the first daratumumab-based regimen and the date of death. Patients without a record of death were censored at the end of follow-up.

Study measures for the overall population were reported using descriptive statistics. As patients initiating daratumumab at different stages of treatment likely had different patterns and outcomes, results were stratified based on the line of therapy at daratumumab initiation (i.e., first line [1 L], second line [2 L], or third line and after [3 L+]).

### Subgroup and sensitivity analyses

Treatment patterns and treatment response outcomes were also reported among patients who were re-treated at least once with daratumumab. Re-treatment was defined as the resumption of a daratumumab-based treatment regimen following a ≥ 90-day period during which daratumumab was not administered. Patients were excluded if they had a stem cell transplant during the gap between the daratumumab-based treatment regimens or during these regimens.

In order to assess more recent treatment patterns, a sensitivity analysis was conducted among patients who initiated daratumumab on or after 2018. Among 1 L patients, this analysis was restricted to patients initiating daratumumab on or after FDA approval for front line treatment (among stem cell transplant recipients: 26 September 2019; non-stem cell transplant: 7 May 2018).

## Results

### Patient characteristics

Among a total of 705 patients who received daratumumab at the two sites between November 2015 and March 2021, 299 patients were included in the study (Levine Cancer Institute: 199; Weill Cornell Medicine: 100). Among them, 26 were 1 L patients, 66 were 2 L patients, and 207 were 3 L+ patients (see Table 1).

**Table 1:** Patient Characteristics

	All patients	Frontline daratumumab patients	Daratumumab initiated in 2 L	Daratumumab initiated in 3 L+	Re-treated patients^**1**^
	***N*** = 299	***N*** = 26	***N*** = 66	***N*** = 207	***N*** = 19
**Demographic characteristics**					
**Age at daratumumab initiation, mean ± SD [median]**	67.7 ± 11.3 [69.0]	68.2 ± 13.9 [72.0]	68.4 ± 10.2 [68.0]	67.4 ± 11.3 [68.0]	67.2 ± 14.7 [66.0]
**Time between MM diagnosis and daratumumab initiation (months), mean ± SD [median]**	35.4 ± 30.6 [29.6]	2.1 ± 1.9 [1.8]	24.2 ± 24.6 [15.1]	43.2 ± 30.4 [36.7]	53.3 ± 41.3 [45.3]
**Sex, n (%)**					
Male	164 (54.8)	15 (57.7)	36 (54.5)	113 (54.6)	10 (52.6)
Female	135 (45.2)	11 (42.3)	30 (45.5)	94 (45.4)	9 (47.4)
**Race, n (%)**					
White	163 (54.5)	15 (57.7)	41 (62.1)	107 (51.7)	10 (52.6)
Black or African American	89 (29.8)	4 (15.4)	11 (16.7)	74 (35.7)	7 (36.8)
Hispanic	6 (2.0)	0 (0.0)	2 (3.0)	4 (1.9)	0 (0.0)
Asian	4 (1.3)	1 (3.8)	1 (1.5)	2 (1.0)	0 (0.0)
Mixed	9 (3.0)	2 (7.7)	3 (4.5)	4 (1.9)	1 (5.3)
Other	1 (0.3)	0 (0.0)	1 (1.5)	0 (0.0)	0 (0.0)
Unknown	27 (9.0)	4 (15.4)	7 (10.6)	16 (7.7)	1 (5.3)
**Primary insurance plan type, n (%)**					
Medicare	181 (60.5)	15 (57.7)	37 (56.1)	129 (62.3)	13 (68.4)
Commercial insurance	46 (15.4)	4 (15.4)	15 (22.7)	27 (13.0)	3 (15.8)
Medicaid	8 (2.7)	0 (0.0)	0 (0.0)	8 (3.9)	0 (0.0)
Other	35 (11.7)	1 (3.8)	7 (10.6)	27 (13.0)	1 (5.3)
Unknown	29 (9.7)	6 (23.1)	7 (10.6)	16 (7.7)	2 (10.5)
**Clinical characteristics**					
**MM stage as of MM diagnosis date (R-ISS)**^**2**^**, n (%)**					
Stage I	58 (19.4)	8 (30.8)	17 (25.8)	33 (15.9)	1 (5.3)
Stage II	104 (34.8)	8 (30.8)	26 (39.4)	70 (33.8)	4 (21.1)
Stage III	58 (19.4)	2 (7.7)	9 (13.6)	47 (22.7)	5 (26.3)
Unknown	79 (26.4)	8 (30.8)	14 (21.2)	57 (27.5)	9 (47.4)
**Cytogenetic profile as of daratumumab initiation**^**3**^**, n (%)**					
Standard	108 (36.1)	19 (73.1)	27 (40.9)	62 (30.0)	9 (47.4)
High	55 (18.4)	4 (15.4)	9 (13.6)	42 (20.3)	2 (10.5)
Unknown	136 (45.5)	3 (11.5)	30 (45.5)	103 (49.8)	8 (42.1)
**Refractory disease prior to daratumumab initiation**^**4**^**, n (%)**					
To any line of therapy prior to daratumumab initiation	210 (76.9)	–	35 (53.0)	175 (84.5)	15 (83.3)
To an immunomodulatory drug	162 (59.3)	–	25 (37.9)	137 (66.2)	10 (55.6)
To a proteasome inhibitor	153 (56.0)	–	14 (21.2)	139 (67.1)	13 (72.2)
To a proteasome inhibitor and an immunomodulatory drug	111 (40.7)	–	7 (10.6)	104 (50.2)	8 (44.4)
**Year of daratumumab initiation, n (%)**					
2015	2 (0.7)	0 (0.0)	0 (0.0)	2 (1.0)	1 (5.3)
2016	32 (10.7)	0 (0.0)	2 (3.0)	30 (14.5)	4 (21.1)
2017	55 (18.4)	0 (0.0)	10 (15.2)	45 (21.7)	4 (21.1)
2018	78 (26.1)	2 (7.7)	20 (30.3)	56 (27.1)	6 (31.6)
2019	90 (30.1)	15 (57.7)	23 (34.8)	52 (25.1)	4 (21.1)
2020	42 (14.0)	9 (34.6)	11 (16.7)	22 (10.6)	0 (0.0)

**Abbreviations:** 2 L: second-line; 3 L: third-line; kg: kilogram; MM: multiple myeloma; R-ISS: Revised International Staging System; SD: standard deviation

Notes

**[1]** Re-treatment was defined as the resumption of a daratumumab-based treatment regimen following a ≥ 90-day period during which daratumumab was not administered. Patients were excluded if they had a stem cell transplant during the gap between the daratumumab-based treatment regimens or during these regimens

**[2]** Definition taken from Palumbo A, Avet-Loiseau H, Oliva S, et al. Revised International Staging System for multiple myeloma: A report from International Myeloma Working Group. *J Clin Oncol* 2015; 33:2863–69

**[3]** Definition taken from Dimopoulos MA, Oriol A, Nahi H, San-Migel J, Bahlis NJ, Usmani S et al. Daratumumab, Lenalidomide, and Dexamethasone for Multiple Myeloma. *New England Journal of Medicine*. 2016; 375 [14]: 1319–1331.; Sonneveld P, Avet-Loiseau H, Lonial S, Usmani S, Siegel D, Anderson KC et al. Treatment of multiple myeloma with high-risk cytogenetics: a consensus of the International Myeloma Working Group. *Blood* 2016; 127 [16]: 2955–2962

**[4]** Refractory disease was established at the regimen level. Refractory disease on a proteasome inhibitor or refractory disease on an immunomodulatory drug was established if a patient’s best response to a treatment regimen including one of these agents was stable disease, progressive disease, or relapse, or if the regimen was discontinued due to disease progression. Refractory disease to a proteasome inhibitor and an immunomodulatory drug was established if a patient’s best response to a treatment regimen including one of these types of agents was stable disease, progressive disease, or relapse, or if the regimen was discontinued due to disease progression

The mean age at daratumumab initiation was 68 years old (median: 69 years, range: 25–93 years) and 164 (54.8%) patients were male. Most patients were either White (163 patients, 54.5%) or Black or African American (29.8%). The proportion of Black or African American patients who initiated daratumumab in 1 L, 2 L and 3 L+ was 15.4, 16.7 and 35.7%, respectively (see Table 1).

The mean time between MM diagnosis and daratumumab initiation was 2.1 months, 24.2 months, and 43.2 months for patients who initiated daratumumab in 1 L, 2 L and 3 L+, respectively. The most common MM stage at diagnosis was Stage II (104 patients, 34.8%); an equal number of patients were Stage I or Stage III (58 patients, 19.4%; see Table 1).

Among the study population, 19 patients were re-treated with daratumumab after their initial daratumumab treatment. These patients had generally similar characteristics at the initiation of daratumumab as compared to the overall sample. However, the mean number of lines of treatment prior to the initiation of the first daratumumab regimen was 3.4, compared with 2.4 for the overall sample (median: 2, range: 0–10).

A total of 206 initiated daratumumab on or after 2018, including 22 1 L patients, 54 2 L patients, and 130 3 L+ patients.

### Treatment patterns of first daratumumab-based regimen

The mean duration of follow-up was 18.4 months (standard deviation [SD]: 12.5) and was shorter among 1 L patients (9.7 months, SD: 6.7) than 2 L (19.2 months, SD: 11.6) or 3 L+ patients (19.3 months, SD: 12.9). Among patients who initiated daratumumab in 1 L, the most common regimens were daratumumab with bortezomib and lenalidomide (± dexamethasone, DVRd, *n* = 12, 46.2%) and daratumumab with lenalidomide (± dexamethasone, DRd, *n* = 9, 34.6%). Among patients who initiated daratumumab in 2 L, the most common regimens were DRd (*n* = 18, 27.3%), daratumumab with bortezomib (± dexamethasone, DVd, *n* = 17, 25.8%) and daratumumab with pomalidomide (± dexamethasone, DPd, *n* = 16, 24.2%). Among patients who initiated daratumumab in 3 L+, the most common regimens were DPd (*n* = 97, 46.9%) and daratumumab monotherapy (± dexamethasone, *n* = 45, 21.7%; see Table 2).

**Table 2:** Treatment Patterns of the First Daratumumab-Based Regimen

	All patients	Frontline daratumumab patients	Daratumumab initiated in 2 L	Daratumumab initiated in 3 L+
	N = 299	N = 26	***N*** = 66	***N*** = 207
**Duration of follow-up**^**1**^ **(months), mean ± SD [median]**	18.4 ± 12.5 [16.6]	9.7 ± 6.7 [7.5]	19.2 ± 11.6 [16.9]	19.3 ± 12.9 [17.2]
**Number of lines of therapy received prior to daratumumab initiation, mean ± SD [median]**	2.4 ± 1.6 [2.0]	0.0 ± 0.0 [0.0]	1.0 ± 0.0 [1.0]	3.2 ± 1.4 [3.0]
**Number of regimens**^**2**^ **received prior to daratumumab initiation, mean ± SD [median]**	3.2 ± 2.0 [3.0]	0.0 ± 0.0 [0.0]	1.8 ± 1.0 [1.0]	4.1 ± 1.7 [4.0]
**First daratumumab regimen**^**2**^**, n (%)**				
DPd	113 (37.8)	0 (0.0)	16 (24.2)	97 (46.9)
Daratumumab (monotherapy)	51 (17.1)	0 (0.0)	6 (9.1)	45 (21.7)
DRd	49 (16.4)	9 (34.6)	18 (27.3)	22 (10.6)
DVd	31 (10.4)	3 (11.5)	17 (25.8)	11 (5.3)
DVMP	0 (0.0)	0 (0.0)	0 (0.0)	0 (0.0)
Other regimens	55 (18.4)	14 (53.8)	9 (13.6)	32 (15.5)
Bortezomib + daratumumab + lenalidomide ± dexamethasone	18 (6.0)	12 (46.2)	6 (9.1)	0 (0.0)
Carfilzomib + daratumumab ± dexamethasone	7 (2.3)	0 (0.0)	0 (0.0)	7 (3.4)
Carfilzomib + daratumumab + pomalidomide ± dexamethasone	3 (1.0)	0 (0.0)	1 (1.5)	2 (1.0)
Daratumumab + ixazomib + pomalidomide ± dexamethasone	2 (0.7)	0 (0.0)	0 (0.0)	2 (1.0)
Other	25 (8.4)	2 (7.7)	2 (3.0)	21 (10.1)
**Received a stem cell transplant prior to initiating daratumumab, n (%)**	110 (36.8)	0 (0.0)	19 (28.8)	91 (44.0)
**Regimen type of daratumumab-based therapy**^**3**^**, n (%)**				
Induction therapy	140 (46.8)	25 (96.2)	32 (48.5)	83 (40.1)
Conditioning therapy	65 (21.7)	1 (3.8)	12 (18.2)	52 (25.1)
Consolidation therapy	23 (7.7)	0 (0.0)	6 (9.1)	17 (8.2)
Maintenance therapy post-stem cell transplant	9 (3.0)	0 (0.0)	1 (1.5)	8 (3.9)
Bridging therapy	2 (0.7)	0 (0.0)	1 (1.5)	1 (0.5)
Unknown/non applicable	80 (26.8)	1 (3.8)	17 (25.8)	62 (30.0)
**Length of regimen (months), mean ± SD [median]**	10.3 ± 10.7 [6.5]	6.9 ± 5.8 [4.1]	12.3 ± 10.5 [8.8]	10.1 ± 11.1 [6.4]

**Abbreviations:** 2 L: second-line; 3 L: third-line; DPd: daratumumab, pomalidomide, and dexamethasone; DRd: daratumumab, lenalidomide, and dexamethasone; DVd: daratumumab, bortezomib, and dexamethasone; DVMP: daratumumab, bortezomib, melphalan, and prednisone; SD: standard deviation

Notes

**[1]** Follow-up was defined as the number of months between the index date and the latest of 1) the end date of the last regimen entered (or date of chart abstraction if the last regimen entered was ongoing at the time of entry), 2) the last recorded best response to a regimen, or 3) death

**[2]** Regimens consisting of the same agents with or without dexamethasone were reported as the same regimen

**[3]** Each daratumumab-based regimen may have > 1 regimen type

A total of 110 patients (36.8%) received a stem cell transplant prior to initiating daratumumab, including 19 patients (28.8%) who had initiated daratumumab in 2 L, and 91 patients (44.0%) who had initiated daratumumab in 3 L+ (see Table 2).

**Fig. 1:**
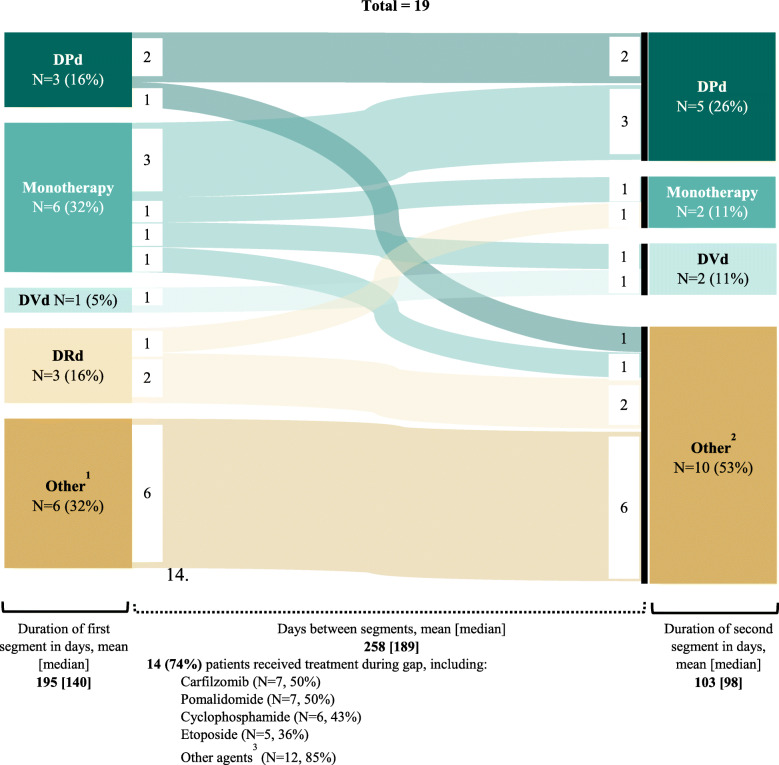
Daratumumab Re-Treatment Patterns. DPd: daratumumab, pomalidomide, and dexamethasone; DRd: daratumumab, lenalidomide, and dexamethasone; DVd: daratumumab, bortezomib, and dexamethasone. **Notes: [1]** Other regimens include: bortezomib + daratumumab + dexamethasone + pomalidomide, bortezomib + cyclophosphamide + daratumumab + dexamethasone, carfilzomib + cisplatin + cyclophosphamide + daratumumab + dexamethasone + etoposide, carfilzomib + daratumumab, carfilzomib + daratumumab + pomalidomide, daratumumab + denosumab + pomalidomide + dexamethasone. **[2]** Other regimens include: bortezomib + daratumumab + dexamethasone + pomalidomide, bortezomib + cyclophosphamide + daratumumab + dexamethasone, carfilzomib + daratumumab, daratumumab + dexamethasone + selinexor, daratumumab + venetoclax, cyclophosphamide + daratumumab + dexamethasone + pomalidomide, carfilzomib + cisplatin + cyclophosphamide + daratumumab + dexamethasone + etoposide + doxorubicin + melphalan + thalidomide, carfilzomib + daratumumab + cyclophosphamide + doxorubicin + etoposide, biaxin + carfilzomib + cisplatin + daratumumab + etoposide + liposomal doxorubicin + venetoclax, DPd + ixazomib.. **[3]** Other agents include: cisplatin, doxorubicin, bortezomib, ixatuzumab, lenalidomide, thalidomide, selinexor, venetoclax, melphalan, panobinostat, and clinical trial/investigational agents

**Among re-treated patients, all but one patient had their first treatment segment in 3 L+. The most common regimen used for the first treatment segment was daratumumab monotherapy (± dexamethasone,** ***n*** **= 6, 31.6%), while the most common regimen used for the second treatment segment was DPd (*n*** **= 5, 26.3%). The mean length of the gap between treatment segments was 258 days (range: 93–644 days). The majority of patients (14/19, 73.7%) had a non daratumumab-based regimen during the ≥ 90-day gap. Six patients (31.6%) remained on the same regimen before and after the ≥ 90-day gap. Among these, four patients did not receive any treatment during the gap. The length of the gap for these four patients ranged between 112 and 195 days. Among the two who did, one patient was treated with bortezomib, dexamethasone and lenalidomide. The other patient received 8 regimens during the gap, 7 of which were carfilzomib-based and 1 of which was an investigational antibody-drug conjugate (see** Fig. 1**).3.3. Clinical outcomes.**

Patients who initiated daratumumab in 1 L, 2 L and 3 L+ had a response rate of 100.0, 78.8 and 65.2%, respectively. The proportion of patients with a “very good partial response” or better was highest among patients initiating daratumumab in 1 L (73.1%), and 57.6 and 40.7% for patients who initiated daratumumab in 2 L and 3 L+, respectively (see Table 3).

**Table 3:** Treatment Response on First Daratumumab-Based Regimen

	All patients	Frontline daratumumab patients	Daratumumab initiated in 2 L	Daratumumab initiated in 3 L+
	N = 299	N = 26	N = 66	N = 207
**Best response achieved per IMWG criteria**^**1**^**, n (%)**				
Stringent complete response	16 (5.4)	5 (19.2)	6 (9.1)	5 (2.4)
Complete response	25 (8.4)	4 (15.4)	4 (6.1)	17 (8.2)
Very good partial response	99 (33.1)	10 (38.5)	28 (42.4)	61 (29.5)
Partial response	71 (23.7)	7 (26.9)	14 (21.2)	50 (24.2)
Minimal response	4 (1.3)	0 (0.0)	2 (3.0)	2 (1.0)
Stable disease	38 (12.7)	0 (0.0)	5 (7.6)	33 (15.9)
Progressive disease	43 (14.4)	0 (0.0)	7 (10.6)	36 (17.4)
Clinical relapse	0 (0.0)	0 (0.0)	0 (0.0)	0 (0.0)
Other	0 (0.0)	0 (0.0)	0 (0.0)	0 (0.0)
Unknown/not available	3 (1.0)	0 (0.0)	0 (0.0)	3 (1.4)
**Patients with known response rate, n (%)**	296 (99.0)	26 (100.0)	66 (100.0)	204 (98.6)
Overall response rate^2^, n (%)	211 (71.3)	26 (100.0)	52 (78.8)	133 (65.2)
Very good partial response or better, n (%)	140 (47.3)	19 (73.1)	38 (57.6)	83 (40.7)
Months from regimen start to best response date, mean ± SD [median]	4.8 ± 5.7 [2.8]	3.7 ± 3.0 [2.9]	5.1 ± 4.9 [3.3]	4.8 ± 6.1 [2.8]

**Abbreviations:** 2 L: second-line; 3 L: third-line; IMWG: International Myeloma Working Group; SD: standard deviation

Notes

**[1]** Kumar S, Paiva B, Anderson KC, Durie B, Landgren O, Moreau P et al. International Myeloma Working Group consensus criteria for response and minimal residual disease assessment in multiple myeloma. Lancet Oncol. 2016 Aug;17 [8]:e328-e346

**[2]** Overall response rate defined as partial response or better among patients with known response rate

Treatment response was similar for patients who initiated daratumumab on or after 2018 (see **Supplementary Table** **1**).

Among re-treated patients, overall response rate was 66.7% during the first treatment segment, and 52.9% during the second treatment segment (see Table 4).

**Table 4:** Treatment Response among Re-Treated Patients

	Daratumumab re-treated patients^**1**^	
	First treatment segment	Second treatment segment
	N = 19	N = 19
**Best response achieved per IMWG criteria**^**2**^**, n (%)**		
Stringent complete response	0 (0.0)	0 (0.0)
Complete response	0 (0.0)	1 (5.3)
Very good partial response	5 (26.3)	2 (10.5)
Partial response	7 (36.8)	6 (31.6)
Minimal response	0 (0.0)	1 (5.3)
Stable disease	5 (26.3)	1 (5.3)
Progressive disease	1 (5.3)	6 (31.6)
Clinical relapse	0 (0.0)	0 (0.0)
Other	0 (0.0)	0 (0.0)
Unknown/not available	1 (5.3)	2 (10.5)
**Patients with known response rate, n (%)**	18 (94.7)	17 (89.5)
Overall response rate^3^, n (%)	12 (66.7)	9 (52.9)
Very good partial response or better, n (%)	5 (27.8)	3 (17.6)
Months from regimen start to best response date, mean ± SD [median]	5.3 ± 4.7 [3.3]	2.0 ± 1.1 [1.8]

**Abbreviations:** IMWG: International Myeloma Working Group; SD: standard deviation

Notes

**[1]** Re-treatment was defined as the resumption of a daratumumab-based treatment regimen following a ≥ 90-day period during which daratumumab was not administered. Patients were excluded if they had a stem cell transplant during the gap between the daratumumab-based treatment regimens or during these regimens

**[2]** Kumar S, Paiva B, Anderson KC, Durie B, Landgren O, Moreau P et al. International Myeloma Working Group consensus criteria for response and minimal residual disease assessment in multiple myeloma. Lancet Oncol. 2016 Aug;17 [8]:e328-e346

**[3]** Overall response rate defined as partial response or better among patients with known response rate

**Fig. 2:**
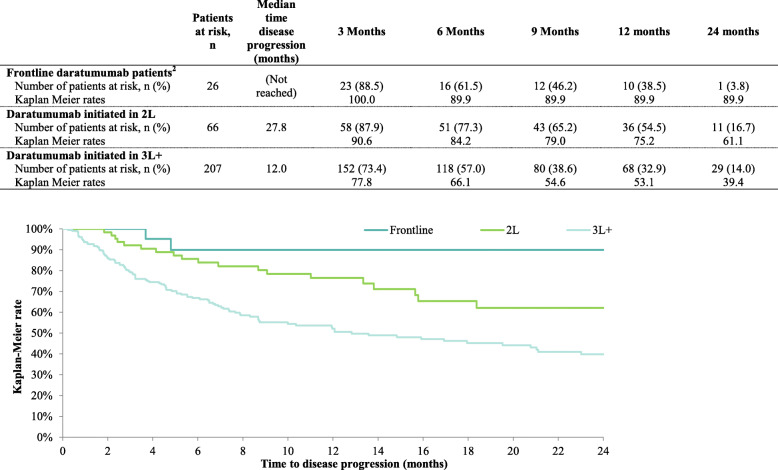
Progression-Free Survival^**1.**^ **Abbreviations:** 2 L: second-line; 3 L: third-line. **Notes: [1]** Disease progression was defined as a record of discontinuation due to progressive disease, progressive disease as a patient’s best response to a treatment regimen, or death and was measured from the index date onward. Patients were censored at the earliest between initiation of a new line of therapy or end of follow-up, whichever occurred fir

The median time to disease progression was not reached among patients who initiated daratumumab in 1 L. Among patients who initiated daratumumab in 2 L, median time to disease progression was 27.8 months. Among patients who initiated daratumumab in 3 L+, median time to disease progression was 12.0 months. Kaplan-Meier rates of PFS at 12 months were 89.8, 75.2 and 53.1% among patients who initiated daratumumab in 1 L, 2 L, and 3 L+, respectively (see Fig. 2).

**Fig. 3:**
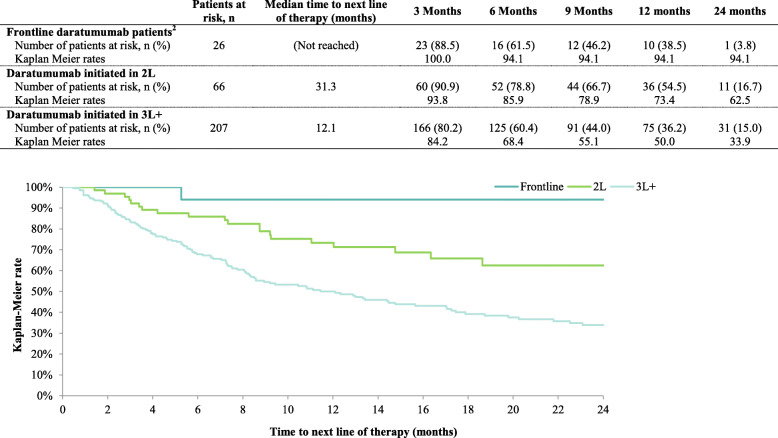
Time to Next Line of Therapy^1.^ **Abbreviations:** 2 L: second-line; 3 L: third-line. **Notes: [1]** Time to next line of therapy was defined as the time between the initiation of the first daratumumab-based regimen (index date) and the initiation of the following line of therapy. Patients not initiating a subsequent line of therapy were censored at the end of follow-up

The median time to next line of therapy was not reached among patients who initiated daratumumab in 1 L. Among patients who initiated daratumumab in 2 L, median time to next line of therapy was 31.3 months. Among patients who initiated daratumumab in 3 L+, median time to disease progression was 12.1 months. Kaplan-Meier rates of time to next line of therapy at 12 months were 94.1, 73.4 and 50.0% among patients who initiated daratumumab in 1 L, 2 L, and 3 L+, respectively (see Fig. 3).

Kaplan-Meier rates of overall survival at 12 months were 93.3, 86.9, and 79.3% among patients who initiated daratumumab in 1 L, 2 L, and 3 L+, respectively, although these results should be interpreted with caution due to the small sample size.

## Discussion

This study reports the treatment patterns of patients initiating daratumumab across several lines of treatment in the real-world in the United States, including in first line and including daratumumab re-treatment. Most other real-world studies have focused on heavily pretreated patients [5, 17–20] or had relatively small sample sizes [21, 22].

In this study, daratumumab was used both in monotherapy and in combination with a variety of different agents. This is consistent with other reports of how daratumumab is used in the real world [23]. While a majority of patients initiated daratumumab in third line or after, the proportion of patients initiating daratumumab in earlier lines increased in the subset of patients initiating in 2018 or later. This is in line with the date of FDA approval of daratumumab for frontline treatment and illustrates a shift in treatment patterns over time.

Overall treatment response observed in this study (1 L: 100.0%, 2 L: 78.8%, 3 L+: 65.2%) show an overall favorable effectiveness profile, notably in earlier lines of treatment. Among patients initiating daratumumab in 1 L, 73% had a very good partial response or better. In recent trials of daratumumab among newly-diagnosed patients, the proportion of patients achieving very good partial response or better ranged from 73 to 83% [10, 11, 24]. However, it is challenging to directly compare these results with the current study, given differences in backbone agents, patient populations (stem cell transplant eligible vs. ineligible) and timing of the treatment response assessment. Nevertheless, the findings from this study provide real-world evidence that daratumumab is an effective treatment option in front line.

This may be of particular relevance, as a recent retrospective analysis of three large US-based database found that approximately 57% of newly-diagnosed patients with MM received only one line of therapy. Therefore, treating patients with the most effective treatment regimens at diagnosis rather than reserving them for later lines of therapy may increase clinical benefit [16].

Among patients initiating daratumumab in later lines of treatment, treatment response and PFS were sustained, despite more advanced stage of MM, the refractoriness of patients who initiate multiple lines of treatment, and the heterogeneity of the treatment patterns [25]. Furthermore, re-treatment with daratumumab appeared to be effective, with a response rate for the second segment comparable for that of the first daratumumab treatment segment (treatment response following second segment: 52.9%). These findings supplement those of a study that found that some patients may respond to DPd even if they had been refractory to a prior exposure to daratumumab and/or pomalidomide [12].

The findings of this study should be interpreted in light of certain limitations. First, data were restricted to what was available in patients’ medical charts and EMR at the treatment sites. Elements of disease history or progression recorded outside of the sites were not captured in this study. Second, clinical outcomes measures including overall survival should be interpreted with caution due to the relatively small sample size and short duration of follow-up. Third, even if data were entered in a standardized eCRF by data abstractors at both sites who had received training on how to use the eCRF, data are subject to data entry mistakes or omissions. Finally, both sites participating in this study are research facilities, both of which are highly experienced treating patients with daratumumab. Therefore, results of this study may not be generalizable to other settings or less experienced clinical centers.

## Conclusion

In this study, patients initiating daratumumab across different lines of therapy had high rates of response and long PFS. Patients treated with front line daratumumab showed the greatest clinical benefit, with response rates comparable to those observed in recent clinical trials of newly-diagnosed patients. Patients initiating daratumumab in later lines and patients re-treated with daratumumab also had high response rates. These findings suggest that daratumumab-based regimens are an effective treatment option across all lines of therapy in real-world practice, with the greatest benefit observed in 1 L.

## Supplementary Information

**Additional file 1: Supplementary Table 1.** Treatment Response on First Daratumumab-Based Regimen among Patients Initiating Daratumumab in 2018 or Later.

## Data Availability

The datasets generated and/or analyzed during the current study are not publicly available due to restrictions but are available from the corresponding author on reasonable request.

## Abbreviations

1 L: First line
2 L: Second line
3 L: Third line or later.
ASCT: Autologous stem cell transplantation
eCRF: Electronic case report form
EMR: Electronic medical record
DPd: Daratumumab plus pomalidomide and dexamethasone
DRd: Daratumumab plus lenalidomide and dexamethasone
DVd: Daratumumab plus bortezomib and dexamethasone
DVRd: Daratumumab plus bortezomib and lenalidomide and dexamethasone
FDA: U.S. Food and Drug Administration
IMWG: International Myeloma Working Group
IRB: Institutional review board
ISS: International Staging System
MM: Multiple myeloma
ORR: Overall Response Rate
PFS: Progression-free survival
SD: Standard deviation
US: United States

## References

[1] Smith D, Yong K (2013). Multiple myeloma. BMJ..

[2] Surveillance Epidemiology and End Results (SEER). Cancer Stat Facts: Myeloma [Available from: https://seer.cancer.gov/statfacts/html/mulmy.html.

[3] Offidani M, Corvatta L, More S, Nappi D, Martinelli G, Olivieri A (2020). Daratumumab for the Management of Newly Diagnosed and Relapsed/refractory multiple myeloma: current and emerging treatments. Front Oncol.

[4] Lonial S, Weiss BM, Usmani SZ, Singhal S, Chari A, Bahlis NJ, Belch A, Krishnan A, Vescio RA, Mateos MV, Mazumder A, Orlowski RZ, Sutherland HJ, Bladé J, Scott EC, Oriol A, Berdeja J, Gharibo M, Stevens DA, LeBlanc R, Sebag M, Callander N, Jakubowiak A, White D, de la Rubia J, Richardson PG, Lisby S, Feng H, Uhlar CM, Khan I, Ahmadi T, Voorhees PM (2016). Daratumumab monotherapy in patients with treatment-refractory multiple myeloma (SIRIUS): an open-label, randomised, phase 2 trial. Lancet..

[5] Usmani SZ, Nahi H, Plesner T, Weiss BM, Bahlis NJ, Belch A, Voorhees PM, Laubach JP, van de Donk NWCJ, Ahmadi T, Uhlar CM, Wang J, Feng H, Qi M, Richardson PG, Lonial S (2020). Daratumumab monotherapy in patients with heavily pretreated relapsed or refractory multiple myeloma: final results from the phase 2 GEN501 and SIRIUS trials. Lancet Haematol.

[6] Lokhorst HM, Plesner T, Laubach JP, Nahi H, Gimsing P, Hansson M, Minnema MC, Lassen U, Krejcik J, Palumbo A, van de Donk NWCJ, Ahmadi T, Khan I, Uhlar CM, Wang J, Sasser AK, Losic N, Lisby S, Basse L, Brun N, Richardson PG (2015). Targeting CD38 with Daratumumab Monotherapy in multiple myeloma. N Engl J Med.

[7] Dimopoulos MA, Oriol A, Nahi H, San-Miguel J, Bahlis NJ, Usmani SZ, Rabin N, Orlowski RZ, Komarnicki M, Suzuki K, Plesner T, Yoon SS, Ben Yehuda D, Richardson PG, Goldschmidt H, Reece D, Lisby S, Khokhar NZ, O’Rourke L, Chiu C, Qin X, Guckert M, Ahmadi T, Moreau P (2016). Daratumumab, Lenalidomide, and dexamethasone for multiple myeloma. N Engl J Med.

[8] Palumbo A, Chanan-Khan A, Weisel K, Nooka AK, Masszi T, Beksac M, Spicka I, Hungria V, Munder M, Mateos MV, Mark TM, Qi M, Schecter J, Amin H, Qin X, Deraedt W, Ahmadi T, Spencer A, Sonneveld P (2016). Daratumumab, Bortezomib, and dexamethasone for multiple myeloma. N Engl J Med.

[9] Chari A, Suvannasankha A, Fay JW, Arnulf B, Kaufman JL, Ifthikharuddin JJ, Weiss BM, Krishnan A, Lentzsch S, Comenzo R, Wang J, Nottage K, Chiu C, Khokhar NZ, Ahmadi T, Lonial S (2017). Daratumumab plus pomalidomide and dexamethasone in relapsed and/or refractory multiple myeloma. Blood..

[10] Mateos MV, Dimopoulos MA, Cavo M, Suzuki K, Jakubowiak A, Knop S, Doyen C, Lucio P, Nagy Z, Kaplan P, Pour L, Cook M, Grosicki S, Crepaldi A, Liberati AM, Campbell P, Shelekhova T, Yoon SS, Iosava G, Fujisaki T, Garg M, Chiu C, Wang J, Carson R, Crist W, Deraedt W, Nguyen H, Qi M, San-Miguel J (2018). Daratumumab plus Bortezomib, Melphalan, and prednisone for untreated myeloma. N Engl J Med.

[11] Moreau P, Attal M, Hulin C, Arnulf B, Belhadj K, Benboubker L, Béné MC, Broijl A, Caillon H, Caillot D, Corre J, Delforge M, Dejoie T, Doyen C, Facon T, Sonntag C, Fontan J, Garderet L, Jie KS, Karlin L, Kuhnowski F, Lambert J, Leleu X, Lenain P, Macro M, Mathiot C, Orsini-Piocelle F, Perrot A, Stoppa AM, van de Donk NWCJ, Wuilleme S, Zweegman S, Kolb B, Touzeau C, Roussel M, Tiab M, Marolleau JP, Meuleman N, Vekemans MC, Westerman M, Klein SK, Levin MD, Fermand JP, Escoffre-Barbe M, Eveillard JR, Garidi R, Ahmadi T, Zhuang S, Chiu C, Pei L, de Boer C, Smith E, Deraedt W, Kampfenkel T, Schecter J, Vermeulen J, Avet-Loiseau H, Sonneveld P (2019). Bortezomib, thalidomide, and dexamethasone with or without daratumumab before and after autologous stem-cell transplantation for newly diagnosed multiple myeloma (CASSIOPEIA): a randomised, open-label, phase 3 study. Lancet..

[12] Nooka AK, Joseph NS, Kaufman JL, Heffner LT, Gupta VA, Gleason C, Boise LH, Lonial S (2019). Clinical efficacy of daratumumab, pomalidomide, and dexamethasone in patients with relapsed or refractory myeloma: utility of re-treatment with daratumumab among refractory patients. Cancer..

[13] Offidani M, Corvatta L, More S, Olivieri A (2021). Novel experimental drugs for treatment of multiple myeloma. J Exp Pharmacol.

[14] Palumbo A, Avet-Loiseau H, Oliva S, Lokhorst HM, Goldschmidt H, Rosinol L, Richardson P, Caltagirone S, Lahuerta JJ, Facon T, Bringhen S, Gay F, Attal M, Passera R, Spencer A, Offidani M, Kumar S, Musto P, Lonial S, Petrucci MT, Orlowski RZ, Zamagni E, Morgan G, Dimopoulos MA, Durie BGM, Anderson KC, Sonneveld P, San Miguel J, Cavo M, Rajkumar SV, Moreau P (2015). Revised international staging system for multiple myeloma: a report from international myeloma working group. J Clin Oncol.

[15] Kumar S, Paiva B, Anderson KC, Durie B, Landgren O, Moreau P, Munshi N, Lonial S, Bladé J, Mateos MV, Dimopoulos M, Kastritis E, Boccadoro M, Orlowski R, Goldschmidt H, Spencer A, Hou J, Chng WJ, Usmani SZ, Zamagni E, Shimizu K, Jagannath S, Johnsen HE, Terpos E, Reiman A, Kyle RA, Sonneveld P, Richardson PG, McCarthy P, Ludwig H, Chen W, Cavo M, Harousseau JL, Lentzsch S, Hillengass J, Palumbo A, Orfao A, Rajkumar SV, Miguel JS, Avet-Loiseau H (2016). International myeloma working group consensus criteria for response and minimal residual disease assessment in multiple myeloma. Lancet Oncol.

[16] Fonseca R, Usmani SZ, Mehra M, Slavcev M, He J, Cote S, Lam A, Ukropec J, Maiese EM, Nair S, Potluri R, Voorhees PM (2020). Frontline treatment patterns and attrition rates by subsequent lines of therapy in patients with newly diagnosed multiple myeloma. BMC Cancer.

[17] Jelinek T, Maisnar V, Pour L, Spicka I, Minarik J, Gregora E (2018). Adjusted comparison of daratumumab monotherapy versus real-world historical control data from the Czech Republic in heavily pretreated and highly refractory multiple myeloma patients. Curr Med Res Opin.

[18] Kumar S, Durie B, Nahi H, Vij R, Dimopoulos MA, Kastritis E, Terpos E, Leleu X, Beksac M, Goldschmidt H, Hillengass J, Su Z, Hutton B, Cameron C, Khan I, Lam A (2019). Propensity score matching analysis to evaluate the comparative effectiveness of daratumumab versus real-world standard of care therapies for patients with heavily pretreated and refractory multiple myeloma. Leuk Lymphoma.

[19] Salomon-Perzynski A, Walter-Croneck A, Usnarska-Zubkiewicz L, Dytfeld D, Zielinska P, Wojciechowska M (2019). Efficacy of daratumumab monotherapy in real-world heavily pretreated patients with relapsed or refractory multiple myeloma. Adv Med Sci.

[20] Beksac M, Aydin Y, Goker H, Turgut M, Besisik SK, Cagirgan S, Tuglular T, Vural F, Yagci M, Alacacioglu I, Aytan P, Goksoy HS, Gulbas Z, Gunes AK, Gurkan E, Hacioglu SK, Karti SS, Kaynar L, Ozdogu H, Paydas S, Solmaz S, Sonmez M, Tekgunduz E, Yildirim R, Ilhan O (2020). Early access program results from Turkey and a literature review on Daratumumab Monotherapy among heavily pretreated patients with relapsed/refractory myeloma. Clin Lymphoma Myeloma Leuk.

[21] Minarik J, Pour L, Maisnar V, Spicka I, Jungova A, Jelinek T, Brozova L, Krhovska P, Scudla V, Hajek R (2019). Single agent daratumumab in advanced multiple myeloma possesses significant efficacy even in an unselected "real-world" population. Biomed Pap Med Fac Univ Palacky Olomouc Czech Repub.

[22] Byun JM, Yoon SS, Koh Y, Kim I, Jo J, Park H (2019). Daratumumab Monotherapy in heavily pretreated Asian patients with relapsed and refractory multiple myeloma: a real-world experience. Anticancer Res.

[23] Madduri D, Hagiwara M, Parikh K, Pelletier C, Delea TE, Kee A, Chari A (2021). Real-world treatment patterns, healthcare use and costs in triple-class exposed relapsed and refractory multiple myeloma patients in the USA. Future Oncol.

[24] Facon T, Kumar S, Plesner T, Orlowski RZ, Moreau P, Bahlis N, Basu S, Nahi H, Hulin C, Quach H, Goldschmidt H, O’Dwyer M, Perrot A, Venner CP, Weisel K, Mace JR, Raje N, Attal M, Tiab M, Macro M, Frenzel L, Leleu X, Ahmadi T, Chiu C, Wang J, van Rampelbergh R, Uhlar CM, Kobos R, Qi M, Usmani SZ (2019). Daratumumab plus Lenalidomide and dexamethasone for untreated myeloma. N Engl J Med.

[25] Lovas S, Varga G, Farkas P, Masszi T, Wohner N, Bereczki A, Adamkovich N, Borbényi Z, Szomor Á, Alizadeh H, Szaleczky E, Wolf K, Schneider T, Plander M, Szendrei T, Csacsovszki O, Csukly Z, Rajnics P, Egyed M, Nagy Z, Rejtő L, Illés Á, Mikala G, Váróczy L (2019). Real-world data on the efficacy and safety of daratumumab treatment in Hungarian relapsed/refractory multiple myeloma patients. Int J Hematol.

