# Correction to: Treatment patterns and effectiveness of patients with multiple myeloma initiating Daratumumab across different lines of therapy: a real-world chart review study

**DOI:** 10.1186/s12885-021-09015-9

**Published:** 2021-12-02

**Authors:** Shebli Atrash, Philippe Thompson-Leduc, Ming-Hui Tai, Shuchita Kaila, Kathleen Gray, Isabelle Ghelerter, Marie-Hélène Lafeuille, Patrick Lefebvre, Adriana Rossi

**Affiliations:** 1 Levine Cancer Institute, Charlotte, NC USA; 2 Analysis Group, Inc, 1190 avenue des Canadiens-de-Montréal, Deloitte Tower, Suite 1500, Montreal, QC H3B 0G7 Canada; 3 Janssen Scientific Affairs, LLC, Horsham, PA USA; 4 Division of Hematology and Medical Oncology, Weill Cornell Medicine, New York, NY USA

**Correction to: BMC Cancer 21, 1207 (2021)**

**https://doi.org/10.1186/s12885-021-08881-7**

Following publication of the original article [1], it was identified that due to typesetting mistakes, an unfinalized version of the article were published. The following changes have been implemented.

1. The corresponding author’s email address has been corrected: Philippe.Thompson-Leduc@analysisgroup.com
2. In table 1, note 2, the full reference is: Palumbo A, Avet-Loiseau H, Oliva S, Lokhorst HM, Goldschmidt H, Rosinol L, et al. Revised international staging system for multiple myeloma: a report from international myeloma working group. J Clin Oncol. 2015;33(26):2863–9. 10.1200/JCO.2015.61.2267
3. In table 2, note 3, the full reference is: Dimopoulos MA, Oriol A, Nahi H, San-Miguel J, Bahlis NJ, Usmani SZ, et al. Daratumumab, Lenalidomide, and dexamethasone for multiple myeloma. N Engl J Med. 2016;375(14):1319–31. 10.1056/NEJMoa1607751
4. In table 3, note 1, the full reference is: Kumar S, Paiva B, Anderson KC, Durie B, Landgren O, Moreau P, et al. International myeloma working group consensus criteria for response and minimal residual disease assessment in multiple myeloma. Lancet Oncol. 2016;17(8):e328–e46. 10.1016/S1470-2045(16)30206-6
5. In table 4, note 2, the full reference is: Kumar S, Paiva B, Anderson KC, Durie B, Landgren O, Moreau P, et al. International myeloma working group consensus criteria for response and minimal residual disease assessment in multiple myeloma. Lancet Oncol. 2016;17(8):e328–e46. 10.1016/S1470-2045(16)30206-6
6. In the section, *Treatment patterns of first daratumumab-based regimen*, paragraph three: The text should not have been in bold typeface.
7. In the section, *Treatment patterns of first daratumumab-based regimen*, paragraph three: “3.3 Clinical outcomes” Was removed from the end of the paragraph.
8. In the abbreviations list, “3 L: Third line or later” was updated to “3 L+: Third line or later”

These corrections were typesetting errors and do not influence the results of this article. The publishers apologise for this error. The original article [1] has been updated.
