# Supplementary material for: Treatment patterns and effectiveness of patients with multiple myeloma initiating Daratumumab across different lines of therapy: a real-world chart review study
Source: BMC Cancer. 2021 Nov 12;21:1207. doi: 10.1186/s12885-021-08881-7 (PMC8590284; doi:10.1186/s12885-021-08881-7)
Supplement: Supplementary file 1 — Additional file 1: Supplementary Table 1. Treatment Response on First Daratumumab-Based Regimen among Patients Initiating Daratumumab in 2018 or Later. [file 12885_2021_8881_MOESM1_ESM.docx]

# ADDITIONAL MATERIAL

**File name:** Supplementary Table 1

**File format:** Word document (.docx)

**Title of data:** Treatment Response on First Daratumumab-Based Regimen among Patients Initiating Daratumumab in 2018 or Later

|  | **All patients** | **Frontline daratumumab patients** | **Daratumumab initiated in 2L** | **Daratumumab initiated in 3L+** |
| --- | --- | --- | --- | --- |
|  | **N= 206** | **N= 22** | **N= 54** | **N= 130** |
| **Best response achieved per IMWG criteria^1^, n (%)** |  |  |  |  |
| Stringent complete response | 10 (4.9) | 2 (9.1) | 5 (9.3) | 3 (2.3) |
| Complete response | 18 (8.7) | 4 (18.2) | 3 (5.6) | 11 (8.5) |
| Very good partial response | 76 (36.9) | 9 (40.9) | 25 (46.3) | 42 (32.3) |
| Partial response | 49 (23.8) | 7 (31.8) | 9 (16.7) | 33 (25.4) |
| Minimal response | 4 (1.9) | 0 (0.0) | 2 (3.7) | 2 (1.5) |
| Stable disease | 26 (12.6) | 0 (0.0) | 5 (9.3) | 21 (16.2) |
| Progressive disease | 22 (10.7) | 0 (0.0) | 5 (9.3) | 17 (13.1) |
| Unknown/not available | 3 (1.5) | 1 (4.5) | 1 (1.9) | 1 (0.8) |
| **Patients with known response rate, n (%)** | 203 (98.5) | 21 (95.5) | 53 (98.1) | 129 (99.2) |
| Overall response rate^2^, n (%) | 152 (74.9) | 21 (100.0) | 42 (79.2) | 89 (69.0) |
| Very good partial response or better, n (%) | 103 (50.7) | 14 (66.7) | 33 (62.3) | 56 (43.4) |
| Months from regimen start to best response date, mean ± SD [median] | 4.4 ± 4.8 [2.8] | 3.5 ± 3.2 [2.8] | 4.8 ± 5.0 [2.9] | 4.4 ± 5.0 [2.8] |

**Abbreviations:** 2L: second-line; 3L: third-line; IMWG: International Myeloma Working Group; SD: standard deviation

**Notes:**

**[1]** Kumar S, Paiva B, Anderson KC, Durie B, Landgren O, Moreau P et al. International Myeloma Working Group consensus criteria for response and minimal residual disease assessment in multiple myeloma. Lancet Oncol. 2016 Aug;17(8):e328-e346.

**[2]** Overall response rate defined as partial response or better among patients with known response rate.
